# Analysis of affordability differences for rare diseases in China: a comparison across disease types and regions

**DOI:** 10.1186/s12939-024-02137-z

**Published:** 2024-03-19

**Authors:** Ye Chen, Xinyang Chen, Yi Deng, Jinxi Ding

**Affiliations:** School of International Pharmaceutical Business, China Pharmaceutical University, Nanjing, China

**Keywords:** Rare diseases, Affordability, Disease type differences, Regional differences

## Abstract

**Background:**

China has implemented policies to make rare diseases more affordable. While previous studies evaluated overall affordability, few have examined affordability differences across regions and disease types. Given the vastness of China and varying medical policies across cities, this study assesses the affordability of rare diseases based on China’s First List of Rare Diseases (CFLRD), National Reimbursement Drug List (NRDL), and outpatient chronic and special disease policies in each prefecture.

**Method:**

Six rare diseases were selected and the average annual treatment cost of all relevant drugs in NRDL was calculated for each disease. Based on the WHO/HAI standardized approach, the study analyzed 289 cities with outpatient chronic and special disease policies, measured the security levels by the actual reimbursement ratio of Basic Medical Insurance (BMI) and affordability by the ratio of individual expenses after reimbursement to the annual disposable income of urban residents in the province. The security levels and affordability differences across disease types and provinces were analyzed using the Mann-Whitney U test and the K-W test.

**Result:**

The affordability of rare diseases varied significantly on the disease types and annual treatment cost. Diseases with an annual treatment cost below 100 000 yuan are affordable to all prefectures even with low reimbursement rates, while those with a higher treatment cost were not affordable in at least 80% of prefectures even though the reimbursement ratio is high. The affordability of the same disease varies significantly across provinces and municipalities. Outpatient chronic and special diseases insurance and critical illness insurance, and the inconsistencies between them, result in regional differences.

**Conclusion:**

Although China has made progress in improving the affordability of rare diseases, significant differences persist between cities and diseases. The study suggests the optimization of the BMI system and explores independent funds and innovative insurance models to enhance the affordability of rare diseases, particularly those with extremely high treatment costs.

## Introduction

Rare diseases are defined as diseases with a very low prevalence. The United States Orphan Drug Act defines a rare disease as one that affects a population of less than 200,000 [1], Europe defines a rare disease as one with a prevalence rate of less than 5/10,000 [2], and Japan defines a rare disease as one with a prevalence of less than 50,000 people [3]. Given the small number of patients with rare diseases and the high investment in research and development, few companies pay attention to drugs for rare diseases. Due to the limited availability of drugs for rare diseases, the cost is often high, resulting in a significant burden for patients affected by these conditions. Alleviating the burden of patients with rare diseases has become a pressing global issue that needs to be addressed by nations worldwide.

Countries such as the United States, Singapore, Australia and Japan have adopted legislation to provide a variety of preferential policies for rare diseases. These policies incentivize the research and development of drugs for rare diseases, and improve insurance coverage for rare diseases patients [4]. In recent years, China has been focusing on rare diseases and has implemented several policies to improve diagnosis and treatment. One such policy is the establishment of a rare disease diagnosis and treatment alliance. Additionally, the government is encouraging research, development, and production of therapeutic drugs for rare diseases, improving access to these drugs for patients, and creating a mechanism to negotiate access to BMI and prioritize drugs for rare diseases [5, 6]. A total of 28 drugs for rare diseases have been included in the NRDL through the price negotiation system since 2017, with an average price reduction of 55.96%. The introduction of a series of policies has improved the availability and affordability of drugs for rare diseases to varying degrees, and the difference in affordability between urban and rural areas has narrowed [7]. But overall the affordability of drugs for rare diseases is still poor, and most drugs for rare diseases are still unaffordable for urban and rural residents in China [8–10].

There have been studies on rare diseases and the availability and affordability of drugs for rare diseases in China, and most of them use the WHO/HAI standardized approach to measure patient affordability by substituting the average annual income of urban and rural residents for the lowest-paid unskilled government worker (LPGW). However, in the selection of data on the burden of disease on patients and the average annual income of the population, a uniform reimbursement policy and the average annual income of the population at the national level are mostly adopted. For example, one study examined the affordability of rare diseases for urban and rural middle-income residents in China at a 5% co-payment rate [8]. Another study calculated patient out-of-pocket costs using the actual average reimbursement rate of 63.2% in Shandong Province in 2020 (with an upfront out-of-pocket ratio of 30% set for Class B drugs) and measured the affordability of rare diseases using the average daily disposable incomes of urban and rural residents in Shandong Province [10]. China’s vastness results in considerable differences in economic development across different regions. Although China is working towards increasing the level of coordination in its healthcare system, the current level remains relatively low and is primarily driven by municipal-level pooling. This results in treatment policies being formulated by individual municipalities, leading to significant differences in outpatient chronic and special diseases reimbursement policies based on the type of disease and the municipality. As a result, there can be considerable variations in actual reimbursement policies for insurance across different municipalities and disease types. Therefore, it is worthwhile to explore whether these factors have led to differences in the affordability of patients suffering from rare diseases.

Based on the fact that China is geographically highly heterogeneous, this paper measures the affordability of rare diseases by utilizing the health insurance policies of the relevant diseases in each prefecture, as well as the annual per capita income of urban residents in each province, and focuses on exploring the affordability of the disease types and the regional variability.

## Methods

### Data sources

#### The selection of diseases

The selection criteria for the sample diseases in this paper are as follows:

First, the scope of the initial selection of rare diseases was determined based on the National Reimbursement Drug List (2022) (NRDL 2022) issued by the National Healthcare Security Administration and China’s First List of Rare Diseases (CFLRD) issued by the National Health Commission (NHC), which means that the disease is in the CFLRD and that the therapeutic medication is available in the NRDL 2022 [11].

Second, the average annual treatment cost of all drugs in NRDL 2022 was used as the treatment cost of the disease, and a random sampling method was used based on the stratification of the disease cost (less than 10,000 yuan, 10,000 yuan − 50,000 yuan, 50,000 yuan − 100,000 yuan, 100,000 yuan − 200,000 yuan, 200,000 yuan − 300,000 yuan, and more than 300,000 yuan) to ultimately select the six diseases, namely, hemophilia, Parkinson Disease (Young-onset, Early-onset) (PD), multiple sclerosis (MS), systemic sclerosis (SSc), Niemann-Pick disease (NPD), and Fabry disease (FD), as the sample diseases.

#### Data sources

The data used in this paper are all public. This paper accounts for the coverage of outpatient chronic and special disease policies in 333 prefectural-level cities nationwide, of which 33 prefectural-level cities’ relevant policy documents are not available and are not included in the statistics. 299 cities were covered by Basic Medical Insurance for Urban and Rural Residents (BMIURR) and 295 cities were covered by Basic Medical Insurance for Urban Employees (BMIUE). The outpatient chronic and special disease policies are all obtained from the official websites of the local municipal healthcare security administrations.

The cost of medicines for patients with rare diseases constitutes a major chunk of their treatment expenses. As it is not possible to determine the exact number of patients who use each medicine, the sales data of each medicine in key cities is the only available source of information. The price and dosage variations among different medicines make it difficult to determine the actual number of people who use them, based solely on the proportion of sales. As a result, it becomes challenging to accurately calculate the weighted average cost of the disease. Therefore, this paper measures the affordability of patients with rare diseases by taking the average annual treatment cost of all medicines used to treat the disease within the BMI as the treatment cost of the disease. Drug prices were obtained from the Yaozhi Database, based on the lowest bid price in the last three years, and the annual treatment cost (Table 1) was calculated based on the daily dosages in the package insert as well as relevant guidelines. The per capita disposable income of the residents of the 31 provinces is based on the data published by the National Bureau of Statistics (NBS) for the year 2022.

**Table 1:** Annual cost of drugs in the NRDL for sample diseases

Rare Disease	Drug	Daily Dose	Duration of a Treatment Course	Price(Yuan)	Annual Treatment Cost (Ten Thousand Yuan)	Average Annual Cost of Treatment(Ten Thousand Yuan)
FD	Agalsidase Alfa Concentrated Solution for Infusion	12 mg	1 × 27	3100(3.5 ml:3.5 mg/vial)	28.70	28.70
NPD	Miglustat	600 mg	365	128(100 mg/capsule)	28.03	28.03
Hemophilia	Recombinant Human Coagulation Factor VIIa for Injection	10.8 mg	1 × 20.5	14467.66(5 mg/vial)	64.06	20.92
	Recombinant Coagulation Factor IX for Injection	1500IU	3 × 13	5963.63(1000IU/vial) 3508.2(500IU/vial)	34.89	
	Recombinant Coagulation Factor IX	1500IU	3 × 13	888(500IU)	10.39	
	Recombinant Coagulation Factor VIII	1500IU	3 × 20.5	889.95(500IU/vial) 1512.92(1000IU/vial)	13.96	
	Human Coagulation Factor VIII	4500IU	1 × 20.5	370(200IU/vial)	17.06	
	Desmopressin Injection	36 µg	2 × 20.5	50.05(15ug/ml/vial)	0.49	
	Human Prothrombin Complex	1500IU	3 × 13	192(200IU/vial)	5.62	
MS	Ofatumumab Injection	20 mg	1 × 12	6778(0.4 ml:20 mg/vial)	8.13	8.00
	Dimethyl Fumarate Enteric Capsules	480 mg	365	60.8(240 mg/capsule)	4.44	
	Fingolimod Hydrochloride Capsules	0.5 mg	365	228(0.5 mg/capsule)	8.32	
	Siponimod Tablets	2 mg	365	49.26(0.25 mg/tablet) 242(2 mg/tablet)	8.83	
	Teriflunomide Tablets	14 mg	365	282(14 mg/tablet)	10.29	
SSc	Nintedanib esilate soft capsules	300 mg	365	126.68(150 mg/capsule);	3.65	3.65
PD	Droxidopa Capsules	600 mg	365	8.53(100 mg/capsule)	1.87	0.67
	Ropinirole Hydrochloride Tablets	3 mg	365	8.145(0.5 mg/tablet)	1.78	
	Rasagiline Mesylate Tablets	1 mg	365	29.5(1 mg/tablet)	1.08	
	Levodopa Tablets	3500 mg	365	0.375(0.25 g/tablet)	0.19	
	Pramipexole Dihydrochloride Tablets	1.5 mg	365	8.38(1.5 mg/tablet)	0.31	
	Entacapone,Levodopa and Carbidopa Tablets	Levodopa,100 mg Carbidopa,25 mg Entacapone,200 mg	365	7.667 (Levodopa 100 mg, Carbidopa 25 mg, Entacapone 200 mg)	0.280	
	Carbidopa and Levodopa Sustained-release Tablets	Carbidopa,125 mg Levodopa,1250 mg	365	1.66 (Carbidopa, 25 mg Levodopa, 250 mg)	0.30	
	Selegiline Hydrochloride Tablets	10 mg	365	2.895(5 mg/tablet)	0.21	
	Amantadine Hydrochloride Tablets	200 mg	365	0.11(0.1 g/tablet)	0.008	

* The dosage of the drug is calculated according to the maintenance dose. The single dose of coagulation factor is based on a moderate bleeding dose of 25 IU/kg, and the duration of treatment is based on the drug insert [12]. The annual number of bleeds for hemophilia A is calculated as 20.5 times, and the number of bleeds for hemophilia B is calculated as 13 [13]. The dose of Siponimod is based on the maintenance dose of CYP2C9 *1/*1, *1/*2, or *2/*2 genotypes, i.e., 2 mg once daily

### Outcome variables

#### The level of disease security coverage

Using the actual reimbursement rate for a disease to measure the level of BMI coverage for that disease. When calculating the actual reimbursement ratio of BMI for the six rare diseases, the reimbursement of outpatient chronic and special diseases as well as the reimbursement of critical illness insurance is taken into account^1^. The average value of the average reimbursement ratio of the prefecture-level cities included in the coverage of outpatient chronic and special diseases is taken as the actual reimbursement ratio of the disease.

$${{\rm{\lambda }}_{\rm{i}}}\,{\rm{ = }}\,{1 \over n}\,\sum\nolimits_{j = 1}^n {{{{O_{ij}}\, + \,{S_{ij}}} \over {{E_i}}}} $$

n is the number of prefecture-level municipalities where disease i is covered by outpatient chronic and special disease insurance.

O = reimbursement from outpatient chronic and special disease insurance.

S = reimbursement from critical illness insurance.

E = average annual drug cost for the disease, based on the average annual cost of treatment for all therapeutic drugs in the NRDL for the disease.

#### The affordability for residents

The affordability is measured by using the adaption of the WHO/HAI standardized approach. The WHO/HAI methodology is based on the calculation of the number of days in a course of treatment for a particular disease for which the cost of the drug is equivalent to the LPGW at a standard dosage of a particular drug [14]. However, due to the vastness of China, the large differences in the level of economic development of different prefectural cities, and the fact that the indicator LPGW is not available in China, this paper refers to the practice of Xin. et al. who adapts the per capita income of Chinese residents as a substitute for LPGW and measure the affordability of medication by the ratio of the annual cost of medication for rare diseases to the average annual per capita income, i.e. the number of years required to work to afford one year’s worth of medication [15] and adjusts the formula of the WHO/HAI standardized approach as follows:

$${Y_{ij}} = {{{E_i}} \over {{I_{ij}}}} = {{{1 \over n}\sum _{r = 1}^n{\rm{(}}365 \times DD{D_{ir}} \times {P_{ir}}\, \div \,{V_{ir}}{\rm{)}}} \over {{I_{ij}}}}$$

i represents the disease, j is the prefecture, r is a particular therapeutic drug, and n refers to the amount of areas included in the calculation.

Y = number of years of work required to pay for annual treatment of the disease.

I = disposable income per capita.

P = minimum retail package price of drugs, based on the lowest national bid price in the last three years.

V = packaged dose of the drug.

DDD = Defined Daily Dose.

If λ ≥ 50%^2^, the actual level of reimbursement is considered high; otherwise, the actual level of reimbursement is considered insufficient. If Y ≤ 1^3^, the patient is considered to be able to afford the medication for the disease; otherwise, the patient is not considered to be able to afford the medication for the disease.

### Statistical analysis

Descriptive statistics were used in this paper. Firstly, statistics were compiled on the types of rare diseases covered by outpatient chronic and special diseases policy, and differences in the number of areas where different diseases were included in outpatient chronic and special disease policy coverage were analyzed. Secondly, the Mann-Whitney U test and K-W test were used to explore whether there are significant differences in the affordability of diseases between different diseases and between different prefecture-level cities. The correlation test was also used to determine the correlation between disease affordability and related factors (e.g., actual reimbursement rate, cost of disease, etc.), and to find the key factors affecting disease affordability.

## Results

### Overall situation

**Fig. 1:**
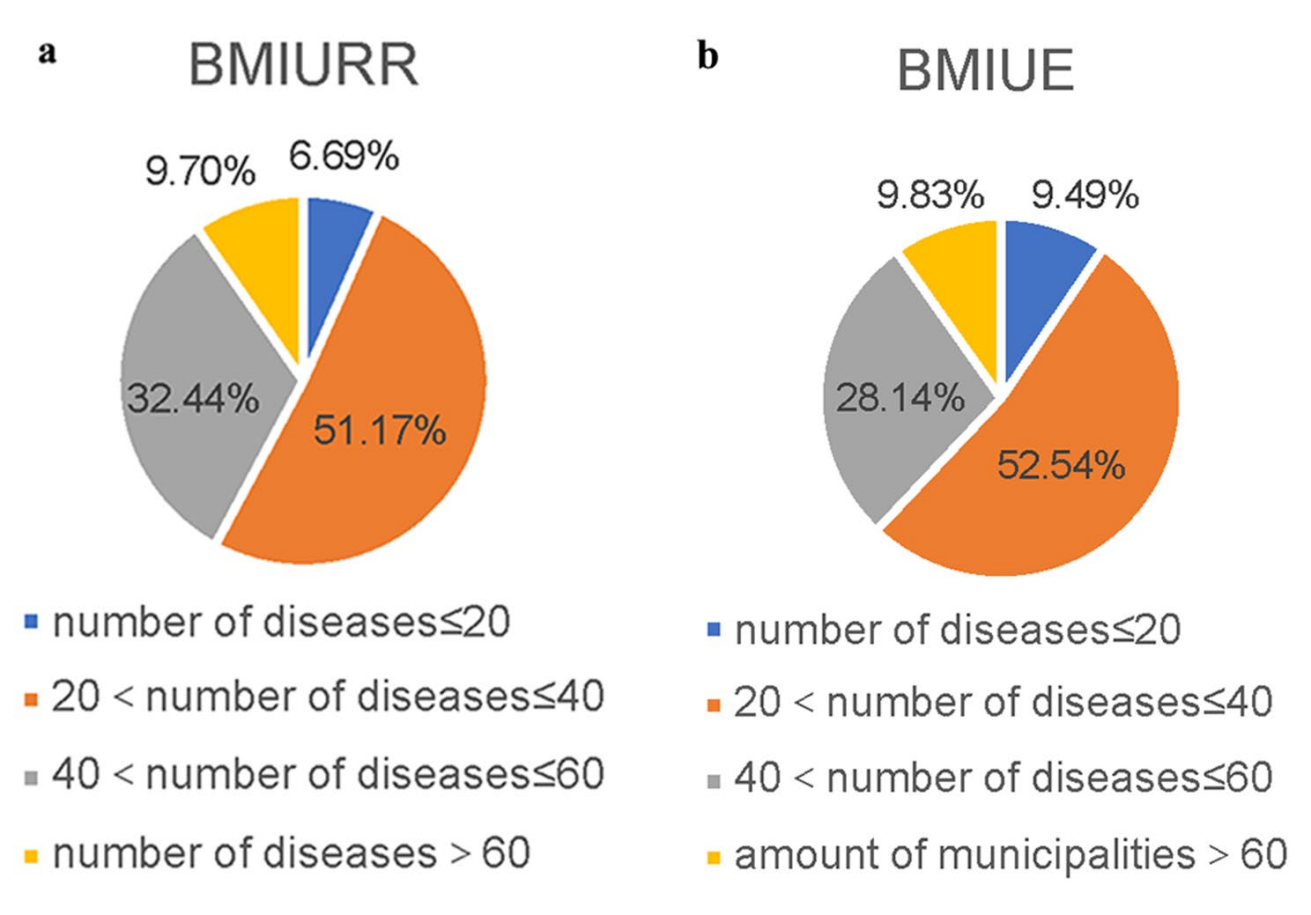
Distribution of the number of diseases covered by the outpatient chronic and special diseases policy by prefecture. **a** distribution of the number of diseases covered by outpatient chronic and special diseases policy of BMIURR by prefecture. **b** distribution of the number of diseases covered by BMIUE outpatient chronic and special diseases policy of BMIUE by prefecture

**Fig. 2:**
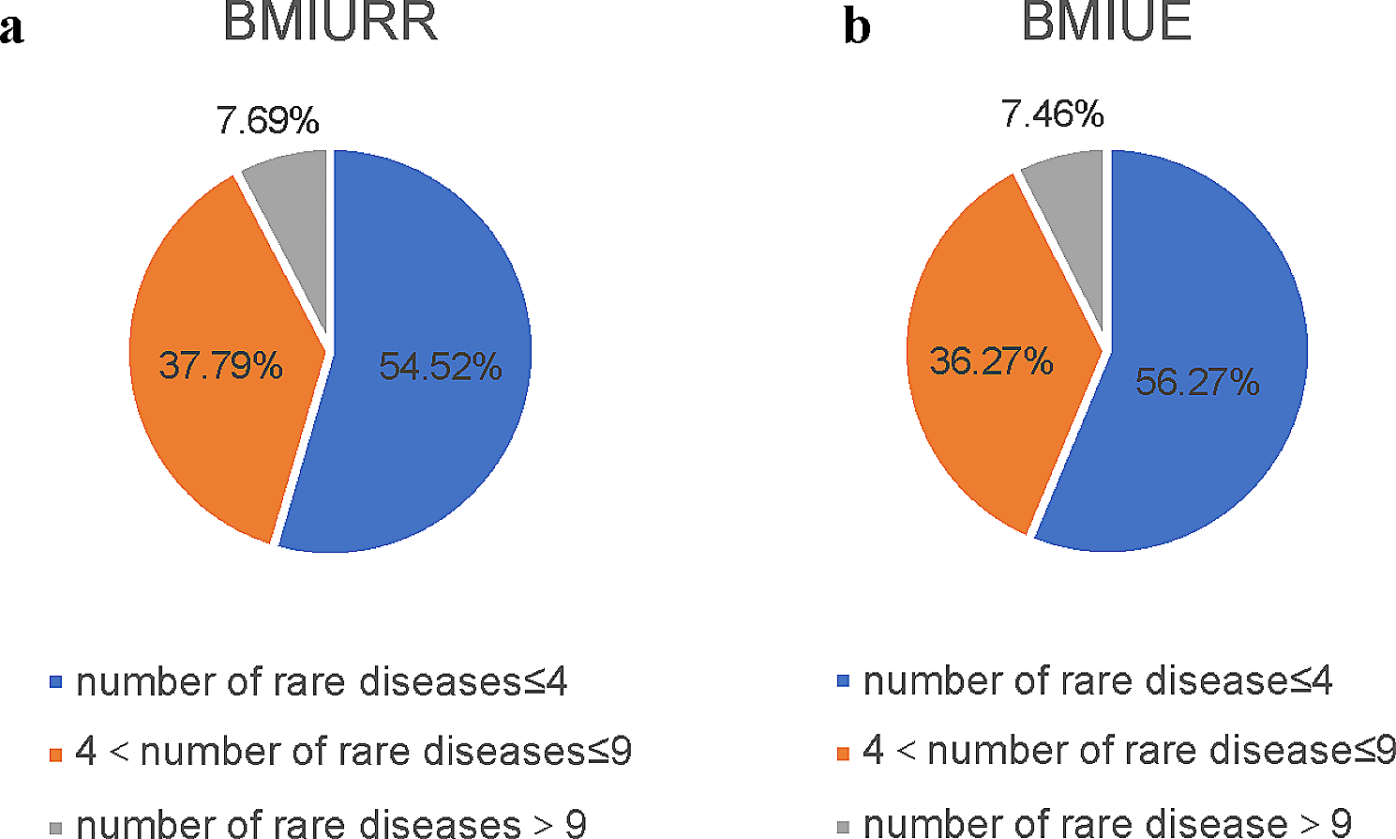
Distribution of the number of rare diseases covered by the outpatient chronic special diseases policy by prefecture. **a** Distribution of the number of rare diseases covered by the outpatient chronic special diseases policy of BMIURR by prefecture. **b** Distribution of the number of rare diseases covered by the outpatient chronic special diseases policy of BMIUE by prefecture

The difference in the average number of chronic and special diseases policy covered for residents and employees is not significant, but there are differences among prefectures: less than 10% of prefectures cover more than 60 diseases, a small number of prefectures cover fewer than 10 diseases, and the majority of prefectures cover 21–40 diseases (Fig. 1). As for the number of rare diseases covered, a total of 24 rare diseases were included in the 300 prefecture-level cities, and most of the prefecture-level cities covered no more than 4 rare diseases (Fig. 2).

The number of diseases covered by the outpatient chronic special disease policy also varies considerably across regions. The most frequently included is hemophilia, which is covered by 284 municipalities, and the least is spinal muscular atrophy, which is covered by only one municipality. There are also large differences in the number of municipalities that have included the 6 sample diseases of hemophilia, PD, MS, SSc, NPD, and FD in their policies for the coverage of outpatient chronic and special diseases for both urban and rural residents and urban employees (Table 2).

**Table 2:** Number of cities where the sample diseases are included in the outpatient chronic and special disease policy

Diseases	BMIURR	BMIUE
hemophilia	279	271
PD	199	205
MS	80	87
SSc	110	113
NPD	32	26
FD	16	16

### Differences in disease types

**Fig. 3:**
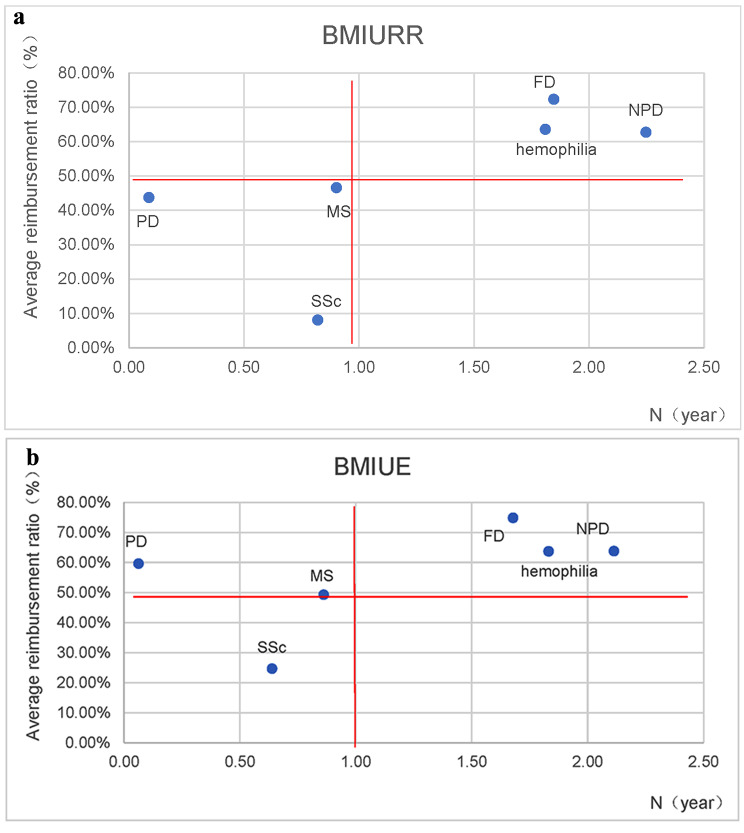
The actual reimbursement rates and affordability for sample diseases. The blue dots represent the affordability of the six diseases respectively. **a** The affordability of BMIURR participants. **b** The affordability of BMIUE participants. * Due to the unavailability of specific reimbursement policies in some municipalities, the final number of municipalities included in the calculation of actual reimbursement rates and years of payment were as follows: 233 for hemophilia BMIURR and 211 for BMIUE, 198 for PD BMIURR and 196 for BMIUE, 53 for MS BMIURR and 60 for BMIUE, 86 for SSc BMIURR and 83 for BMIUE, 9 for FD BMIURR and 9 for BMIUE, and 38 for NPD BMIURR and 30 for BMIUE

The average years of payment required and the average reimbursement rate for each of the six rare diseases separately were calculated, and a scatterplot was drawn (Fig. 3). It was discovered that after being reimbursed by the outpatient chronic special disease policy, for urban and rural insured patients, although the actual reimbursement rate for PD, SSc, and MS was less than 50%, the number of years required to pay was less than 1 year and the diseases were affordable, and the three diseases of hemophilia, FD and NPD, although the actual reimbursement rate was significantly higher than the three diseases mentioned above and exceeded 50%, the number of years required to pay was more than 1 year and are still not affordable.

For urban employees, the average number of years required to pay and the average reimbursement rate displayed similar results. The Mann-Whitney U test result shows that in terms of the same disease, there is a significant difference in affordability between rural and urban residents and urban employees for two diseases, hemophilia and PD (*P* = 0.005, *P* = 0.000), and no significant difference in affordability between residents and urban employees for 4 diseases: MS, SSc, NPD, and FD(*P* = 0.174, *P* = 0.349, *P* = 0.394, *P* = 0.139).

The K-W test was used to analyze the actual reimbursement rate and the payment years for each disease, and the results are shown in Table 3. It shows that there is a significant difference between the actual reimbursement rate and the payment years for different diseases (*P* < 0.05). The highest average reimbursement ratio for both employee’s and residents’ insurance was for NPD, with an average reimbursement ratio of more than 70%, and the lowest was for SSc. But the highest payment years for employees was for FD, for residents was for hemophilia, and the lowest was both for PD. This shows that the actual level of BMI coverage varies greatly between different rare diseases, and that there are large differences in affordability for insurance participants.

**Table 3:** Differences in average actual reimbursement rates and the payment years for different diseases

Items	Type	hemophilia	PD	MS	SSc	NPD	FD	H	P
Average Actual Reimbursement Rates	BMIURR	63.56%	43.75%	46.57%	8.03%	72.32%	62.75%	258.401	0.000
	BMIUE	63.72%	59.60%	49.24%	24.86%	74.86%	63.80%	123.124	0.000
Payment years	BMIURR	1.81	0.09	0.90	0.82	2.25	1.85	504.077	0.000
	BMIUE	1.83	0.06	0.86	0.64	2.11	1.68	452.078	0.000

### Differences in areas

**Fig. 4:**
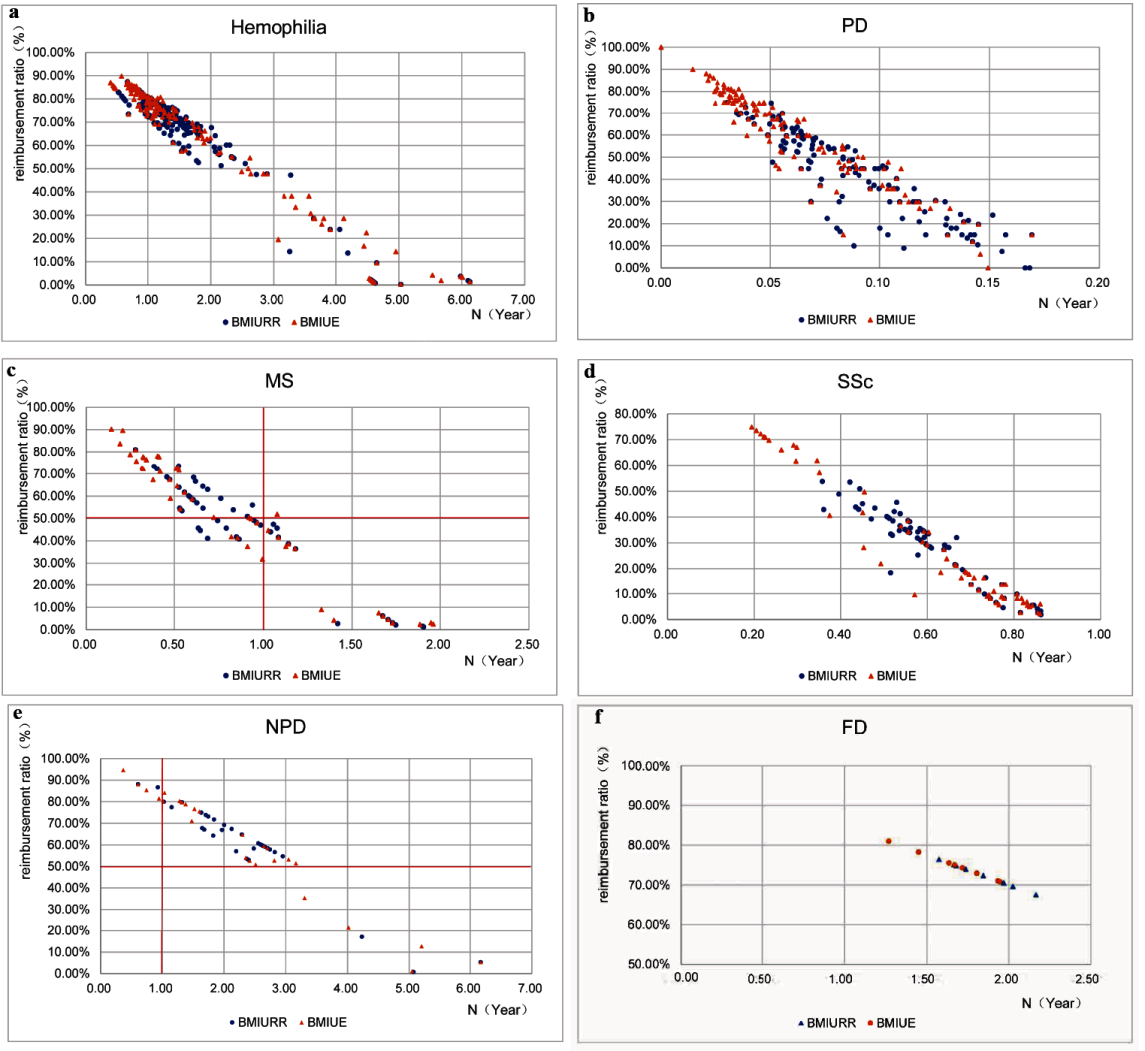
Regional distribution of actual reimbursement ratio and affordability of sample diseases. The blue and orange dots represent the actual reimbursement ratio and payment years of BMIURR and BMIUE for the cities selected respectively. **a-f** The actual reimbursement ratio and payment years of 6 sample diseases

The average reimbursement ratio and the payment year in each municipality after the inclusion of the six rare diseases in the outpatient chronic and special disease coverage were calculated and a scatter plot was made (Fig. 4). It was found that there was a significant negative correlation between the number of years patients needed to work and the actual reimbursement ratio (correlation coefficient = -0.9622), and no strong correlation with the per capita disposable income of the residents of that local municipality (correlation coefficient = -0.3837).

Using the K-W test to analyze the actual reimbursement ratio and the payment years in different provinces and cities, it was found that (Table 4) there are significant differences between the actual reimbursement ratios and the payment years in different provinces for all diseases (*P* < 0.05). Taking hemophilia patients with BMIURR as an example, the actual reimbursement rate can be as high as 87.36% and as low as 0.33%, and the least payment year is only 0.66 years, while the highest can be up to 5.03 years. The actual reimbursement rates and the number of years of work required for residents of NPD were overwhelmingly unaffordable, and there were significant differences between the provinces and cities. PD and SSc are affordable for patients in all provinces and municipalities, although there are significant differences in the actual reimbursement rate and the payment years in each province and municipality.

**Table 4:** Regional Differences in Actual Reimbursement Rates for Sample Diseases

Disease Type	Insurance Type	Actual Reimbursement Rate		Payment Years	
		*H*	*P*	*H*	*P*
hemophilia	BMIURR	129.306	0.000	155.277	0.000
	BMIUE	137.791	0.000	144.621	0.000
PD	BMIURR	76.967	0.000	102.544	0.000
	BMIUE	115.260	0.000	128.200	0.000
MS	BMIURR	27.288	0.000	34.413	0.000
	BMIUE	35.851	0.000	43.389	0.000
SSc	BMIURR	66.144	0.000	70.763	0.000
	BMIUE	66.215	0.000	69.039	0.000
NPD	BMIURR	9.649	0.047	13.201	0.010
	BMIUE	11.897	0.008	12.209	0.007

(* Fabry disease is included in outpatient chronic and special diseases policy in fewer prefectures, which does not allow for significance testing)

## Discussion

The affordability of different diseases presents two features: first, the actual reimbursement rate is not high but still affordable for patients, and second, the actual reimbursement rate is high but still unaffordable for patients, and the cost of disease treatment is an important reason for the difference. Statistics reveal that the average annual drug cost of the former situation is relatively low which is less than 100,000 yuan. Taking systemic sclerosis as an example, there is only one drug for the treatment of systemic sclerosis in the NRDL: Nintedanib esilate soft capsules, with an annual treatment cost of 36,500 yuan. A total of 86 prefectures in China currently include it in the scope of the outpatient chronic and special disease coverage of BMIURR, of which 93.02% of the prefectures have actual reimbursement ratios of less than 50%. However, after being reimbursed by the outpatient chronic and special disease coverage, the payment years are reduced to no more than 1 year for participants in all cities. However, in the case of high-spending rare diseases, the coverage of BMI is insufficient. In the case of hemophilia, for example, there are a total of six types of drugs for hemophilia under BMI, with an average annual treatment cost of 188,100 yuan. A total of 233 prefecture-level cities in China have included hemophilia in the coverage of outpatient chronic and special diseases under the urban residence insurance scheme, and the actual reimbursement rate of the urban residence insurance scheme is higher than 50% in 87.12% of those cities; however, after the reimbursement of the outpatient chronic and special diseases policy, the payment year for the out-of-pocket expenses is still higher than 1 in 86.70% cities.

Therefore, controlling drug prices and lowering the cost of disease treatment through multiple approaches is important for improving affordability. Negotiations on drug prices initiated by the National Health Security Administration (NHSA) to achieve strategic purchasing through health insurance reimbursement eligibility in exchange for better prices is the main way to control the cost of drugs for rare diseases in China. However, the prices of drugs for rare diseases are influenced by a variety of factors, including costs and the market competitive environment. Under the current circumstances, the costs of some drugs for rare diseases cannot be further lowered, and other ways are needed to improve the affordability of the diseases.

Further analysis of the affordability of high-spending rare diseases found that the actual reimbursement rate and patient affordability varied greatly in different regions and that differences in health insurance treatment policies and differences in the payment capacity of BMI are important reasons for the large differences in patient affordability in different regions. Using hemophilia as an example, a comparison of the treatment policies in prefecture-level cities with actual reimbursement ratios above and below 50% shows that 93.33% of the cities with reimbursement ratios below 50% only cover hospitalization costs. The critical illness insurance fails to coordinate with outpatient chronic and special diseases policy in these cities. As a refinement and extension of the BMI system, critical illness insurance is an important policy for reducing the burden on patients with severe diseases. Numerous studies have shown that critical illness insurance can effectively alleviate patients’ out-of-pocket expenses and reduce the probability of poverty arising from illness and returning to poverty due to illness, especially for chronic and critical diseases and for low-income and middle-class people [16, 17]. If the docking of critical illness insurance is not realized, patients will not be able to enjoy the secondary reimbursement of critical illness insurance after outpatient chronic and special disease coverage, resulting in higher out-of-pocket costs for patients and poorer disease affordability. Although the remaining two prefectural-level cities have realized the interface between critical illness insurance and outpatient chronic and special disease insurance, the reimbursement capacity of outpatient chronic and special disease insurance is insufficient, and the threshold for major disease insurance is high or the reimbursement rate is not high, resulting in poorer affordability for the patient after the secondary reimbursement of critical illness insurance.

For the above-mentioned municipalities, there is a need to further improve the BMI policy. Not all serious illnesses require hospitalization, and the wide disparity between outpatient and inpatient reimbursement policy will raise the moral hazard that patients will prefer hospitalization when they only need outpatient treatment [18]. Therefore, it is necessary to first optimize the articulation between critical illness insurance and BMI, realize the good match between critical illness insurance and outpatient chronic and special disease insurance policy, and take the cost of disease treatment as a measure of whether or not to enjoy the secondary reimbursement, to give full play to the role of major disease insurance in “ensuring the critical illnesses”. At the same time, it prevents patients from being transferred from outpatient to hospitalization to obtain a higher level of reimbursement which could have resulted in an unnecessary waste of medical resources. In addition, studies have shown that the role of BMI for urban employees and urban and rural residents is more important in easing the burden of medical care than that of critical illness insurance [19]. Therefore, it is necessary to give priority to optimizing the outpatient chronic and special disease policy, and to achieve a tilt toward the protection of special diseases by differentiating the outpatient chronic and special disease ceiling for different types of diseases, so as to strengthen the capacity of BMI. In the case of critical illness insurance, its important parameters, especially the threshold, can be adjusted to give full play to its role in protecting against serious illnesses [20].. For cities where the actual reimbursement rate is higher than 50%, it is more difficult to further increase the actual reimbursement rate due to the limitations of the BMI’s payment capacity. Therefore, attempts can be made to explore innovative payment modes such as commercial medical insurance and independent funds, so as to accurately reduce the burden of medication on special populations without affecting the operation of the BMI fund. At present, many countries in the world have established independent funds, such as Australia’s Life-Saving Drugs Program (LSDP), Russia’s Expensive Disease Program (EDP), Japan’s Initiative on Rare and Undiagnosed (IRUD), China Taiwan’s Rare Disease Relief Fund (RDRF) [21–24]. To provide separate coverage for patients with rare diseases, especially those that are untreatable costly, and unaffordable to patients, through independent fund-raising.

## Conclusion

Although China has increasingly emphasized the medication coverage of rare disease groups, the affordability of rare disease patients is still characterized by significant differences in disease types and regions. For rare diseases with high treatment costs, the level of BMI coverage is still insufficient and unaffordable for most patients, and it is necessary to optimize BMI or explore innovative payment models to further improve the affordability of medication for patients with rare diseases.

This study has counted the reimbursement policies of 333 prefectural-level cities across the country and calculating the affordability of patients based on them. This approach is more aligned with the reality of China’s low coordination level of basic medical insurance, which is still based on municipal pooling. The study also considers the inconsistency of policies in different areas, providing a more realistic reflection of the current level of treatment in China concerning regional disparities.

This study is certainly not without limitations. Firstly, the research substitutes drug costs for total healthcare costs. This may cause an overestimation of the affordability of the disease since the true healthcare costs of patients with the disease are not available. Secondly, when calculating patient affordability, a modification of the WHO/HAI survey method by using the average disposable income of the region was adopted. The average disposable income of the region may differ from the average disposable income of actual disease patients’ families. What’s more, it would be advantageous to conduct additional surveys of disease patients in the future to more accurately evaluate the regional discrepancies in the affordability of rare diseases in China. This will aid in determining the actual family income and total medical expenses. We kindly advise exercising prudence when interpreting our findings in light of the acknowledged limitations.

## Data Availability

The datasets analyzed in the current study are all available on the official websites of local municipal healthcare security administrations, the Yaozhi Database, and the National Bureau of Statistics.

## Footnotes

1 China’s basic medical insurance policy can be divided into two categories: inpatient and outpatient. Outpatient chronic and special diseases policy is one of the basic medical insurance outpatient reimbursement policies, with each city selecting specific diseases and medicines and formulating a separate reimbursement policy for that type of disease and medicine. Critical illness insurance provides secondary reimbursement for high medical expenses, after reimbursement by basic medical insurance. Different regions have different reimbursement policies on critical illness insurance benefits for residents and employees: in some regions, critical illness insurance is only interfaced with hospitalization, i.e. outpatient expenses are not eligible for secondary reimbursement by it after reimbursement by basic medical insurance; in other regions, the critical illness insurance can be connected with the outpatient chronic and special disease policy, i.e., after reimbursement of outpatient expenses for outpatient chronic disease, patients can enjoy the secondary reimbursement of the critical illness insurance. However, outpatient expenses for diseases and medicines that are not covered by outpatient chronic and special disease policy are not eligible for secondary reimbursement under the critical illness insurance after reimbursement by basic medical insurance.

2 50% is the basic medical insurance reimbursement rate used by the National Healthcare Security Administration to measure the BIA of drugs in the negotiation of the NRDL, which can represent the general situation of the current actual reimbursement rate for outpatient services in China.

3 The WHO/HAI standardized survey method was initially used for the affordability evaluation of essential medicines, which was considered affordable to patients when the cost of the medication regimen was divided by the lowest paid unskilled government worker (LPGW) was less than or equal to one, i.e., when the cost of the medication regimen could be covered by working for one day. Xin et al. have modified the equation and used the per capita income of China instead of LPGW to calculate the ratio of the annual cost of medication for rare diseases to the per capita annual income, i.e., how many years of work are needed to afford one year of treatment with rare disease medications, as a measure of the affordability of medications for rare diseases, and followed the criterion of Y ≤ 1 as the defining criterion of affordability of the disease. In addition, according to the data of the seventh National Census in 2020, the average population per household in China is 2.62. When Y = 1, the cost of rare disease treatment accounts for about 38% of a household’s disposable income, which is close to the WHO standard of judging a household’s catastrophic health expenditure at 40%, and it is reasonable to judge the affordability of a disease by Y ≤ 1.

## Abbreviations

CFLRD: China’s First List of Rare Diseases (2018)
NRDL: National Reimbursement Drug List
MI: Basic Medical Insurance
NHC: National Health Commission of the PRC
PD: Parkinson Disease (Young-onset, Early-onset)
MS: Multiple Sclerosis
SSc: Systemic Sclerosis
NPD: Niemann-Pick Disease
FD: Fabry Disease
NBS: National Bureau of Statistics
BMIURR: Basic Medical Insurance for Urban and Rural Residents
BMIUE: Basic Medical Insurance for Urban Employees
DDD: Daily Defined Dose
